# Setting health research priorities using the CHNRI method: I. Involving funders

**DOI:** 10.7189/jogh.06.010301

**Published:** 2016-06

**Authors:** Igor Rudan, Sachiyo Yoshida, Kit Yee Chan, Simon Cousens, Devi Sridhar, Rajiv Bahl, Jose Martines

**Affiliations:** 1 Centre for Global Health Research, The Usher Institute for Population Health Sciences and Informatics, The University of Edinburgh, Scotland, UK; 2 Department of Maternal, Newborn, Child and Adolescent Health, World Health Organization, Geneva, Switzerland; 3 London School of Hygiene and Tropical Medicine, London, UK

In 2007 and 2008, the World Health Organization's Department for Child and Adolescent Health and Development (later renamed as WHO MNCAH – Maternal, Newborn, Child and Adolescent Health) commissioned five large exercises to define research priorities related to the five major causes of child deaths for the period up to the year 2015. The exercises were based on the CHNRI (Child Health and Nutrition Research Initiative) method, which was just being introduced at the time [1,2]. The selected causes were childhood pneumonia, diarrhoea, birth asphyxia, neonatal infections and preterm birth/low birth weight [3–7]. The context for those exercises was clearly defined: to identify research that could help reduce mortality in children under 5 years of age in low and middle income countries by the year 2015. The criteria used in all five exercises were the “standard” CHNRI criteria: (i) answerability of the research question; (ii) likelihood of the effectiveness of the resulting intervention; (iii) deliverability (with affordability and sustainability); (iv) potential to reduce disease burden; and (v) effect on equity [3–7].

The five criteria used by the scorers were intuitive as they followed the path from generating new knowledge to having an impact on the cause of death. They were chosen with a view to identifying research questions that were most likely to contribute to finding effective solutions to the problems. However, after the five exercises – all of which were published in respected international journals [3–7] – the WHO officers were left with an additional question: how “fundable” were the identified priorities, ie, how attractive were they to research funders? More specifically, should another criterion be added to the CHNRI exercises, which would evaluate the likelihood of obtaining funding support for specific research questions?

To answer these questions, coordinators of the CHNRI exercises at the WHO agreed that it would be useful to invite a number of representatives from large funding organizations interested in child health research to take part in a consultation process at the WHO. The process aimed to explore funders perspectives in prioritization of health research. The funders would be presented with the leading research priorities identified through the CHNRI exercises and asked to discuss any potential variation in their likelihood of being funded. If all the leading priorities were equally attractive to funders and likely to attract funding support, this would indicate that the “standard” CHNRI criteria were sufficient for the process of prioritization. However, if there were large differences in attractiveness of the identified research priorities to funders, then adding another criterion to the exercise – “likelihood of obtaining funding support”, or simply “fundability” – would be a useful addition to the standard CHNRI framework.

## THE MEETING WITH THE FUNDERS (GENEVA, 27–29 MARCH 2009)

In March 2009, MNCAH invited 40 representatives from funding organizations, including the Bill and Melinda Gates Foundation, the Wellcome Trust, National Institutes of Health USA, Department for International Development UK, Save the Children, INCLEN, EPICENTRE, UNICEF, USAID, PATH, Ministry of Science and Technology of India, Ministries of Health of Zambia, Pakistan and Brazil, Global Forum for Health Research, Trinity Global Support Foundation, Children's Investment Fund Foundation, Osaka Research Institute for Maternal and Child Health. Eventually, 16 representatives of funding agencies agreed to take part in the exercise under the condition of anonymity. Moreover, it was understood that their input would not necessarily be the official position of their respective funding agencies, nor would it create any form of funding obligation.

Having explained the aims of the consultation meeting to the representatives of funding agencies, the 16 participants were presented with a list of the top 10 research priorities for each of the five major causes of child deaths: pneumonia, diarrhea, birth asphyxia, neonatal infections and preterm birth/low birth weight [3–7]. This set of 50 research priorities represented roughly the top 5% of all the research ideas submitted for scoring during the CHNRI exercises. The WHO coordinators (RB and JM) explained each of the 50 leading research priorities to the 16 donor representatives. Then, the 16 donor representatives were provided with the list of research priorities and asked to individually identify those that were most likely to receive funding support from their respective organizations.

**Figure 1:**
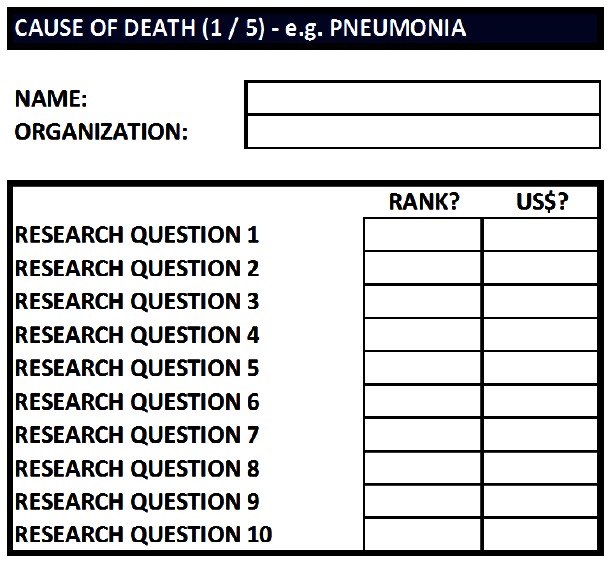
A questionnaire that was given to 16 funder representatives at the meeting to obtain information useful to understanding funding attractiveness of different research priorities.

*Funding attractiveness* was measured in two ways. First, funder representatives were asked to rank the identified research priorities according to their likelihood to receive funding support under an organization’s current investment policies and practices. Second, funding attractiveness was measured by asking funder representatives to distribute a theoretical US$ 100 among the research priorities that seem most fundable. Results were used to facilitate discussion on what makes a research question attractive (or unattractive) for funding support. The scoring sheet that was given to meeting participants is shown in **Figure 1**. While they did not need to provide their name or organization, they were asked to assign ranks 1–10 to the ten research priorities identified for each of the five causes of death (column 1), and also to distribute a hypothetical US$ 100 to different research priorities in concordance to the likely funding support that they may obtain.

Sixteen participants scored the identified research priorities according to the instructions (**Figure 1**). The average ranks across the 16 participants (1 = most likely to be funded; 10 = least likely to be funded) assigned to the 50 research priorities ranged from 3.7 to 7.2. The average US$ amount assigned to research priorities ranged from US$ 20.1 to US$ 2.5. There was general consistency between ranks and the US$ assigned to research priorities.

**Figure 2:**
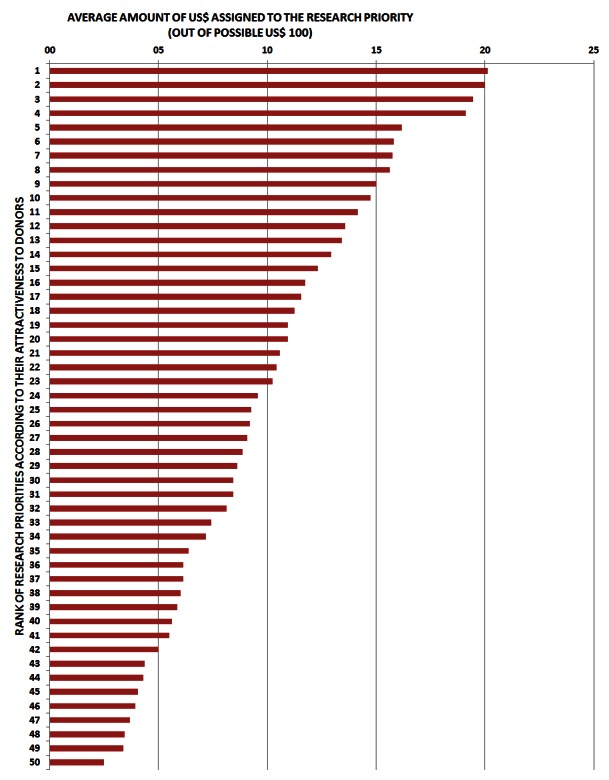
The results of the collective input from 16 funder representatives, showing large differences in funding attractiveness between 50 research priorities. No substantial differences in funding attractiveness would be indicated by equality of the scores on the horizontal axis at the US$ 10.0 line.

Importantly, the analysis of the collective input based on the 2nd column (ie, assigned US$), presented in **Figure 2**, clearly shows that there was a rather substantial departure of the assigned funds from that expected at random: if all research priorities were equally likely to obtain support from the funders, then all the bars would be extending only to the line that represents an investment of US$ 10.0. Furthermore, 4 research priorities (8%) clearly stood out from the rest [8]. It was agreed that they might provide a starting point from which MNCAH Department could concentrate its efforts. These 4 research priorities are shown in **Table 1**.

**Table 1:** The 4 research priorities (8%) that were identified as positive outliers in terms of their likelihood to obtain funding support

Evaluate the quality of community workers to adequately assess, recognize danger signs, refer and treat acute respiratory infections (ARI) in different contexts and settings.
What are the barriers against appropriate use of oral rehydration therapy?
What are the feasibility, effectiveness and cost of different approaches to promote the following home care practices (breastfeeding, cord/skin, care seeking, handwashing)?
What are the feasibility, effectiveness and cost of a scheme of routine home visits for initiation of supportive practices, detection of illness and newborn survival?

## AN ANALYSIS OF THE EXERCISE WITH FUNDER REPRESENTATIVES

The results were analysed after the first day of the meeting and presented to donor representatives at the beginning of the second day of the meeting. An open discussion was held with participants to understand and interpret the results of their collective input. Participants agreed that the most important criteria for research prioritisation differed between researchers and funders. Researchers tended to value answerability, effectiveness, deliverability, impact on the burden and equity. Funders were also interested in the clarity and specificity of research ideas, value for money, novelty, international competitiveness of the groups proposing the research, linkages to broader societal issues, and complementarity with other long–term strategic investments that were already made. An important point in the discussion was that researchers and research funders, especially those in the private sector, often speak quite different languages. Researchers need to be clear on what their goals are and communicate these in more readily understood terms. This point is particularly important because it implies that the CHNRI exercises' research priorities that were identified as most likely to generate useful new knowledge may not be considered equally relevant by the funders. This should certainly be taken into account when presenting and discussing the results of the CHNRI exercises.

Moreover, there seem to be important differences between the categories of funders in the criteria that they use to decide on research priorities. Generally, all investors in health research are concerned with answerability of the proposed research ideas in an ethical way, feasibility and value for money. However, some may be particularly interested in potential for forming partnerships between researchers and industry to increase the translation of findings and their application. Ministries and international organizations appeared more interested in deliverability, affordability and sustainability of the resulting interventions, local and national research capacities to carry out the proposed research ideas, and whether a research question is linked to an ongoing public debate or an important societal issue. Industrial donors may be primarily motivated to generate patents and translate research results into commercial products. Finally, society as a whole may be more concerned with issues of safety and equity and ask whether implementation of research results would widen the existing socio–economic gaps.

Transparency of research priority setting processes must, therefore, begin with those who invest. Perceived returns on investments in health research should be clearly stated at the beginning of the process. They may be defined as reduction in disease burden wherever public money is being invested. Investors from industries may see patentable products as their preferred returns. Non–profit organizations may be primarily interested in increased media attention for their agenda. The context in which investment prioritization takes place is thus primarily defined by expected returns of the funders. Moreover, their investment styles may be balanced and responsible (suggested for those investing public funds), risk–averting (which may be preferred among some industrial partners) or risk–seeking and biased towards high risk – high profit avenues of health research (which may be typical for some industry and not–for–profit organizations).

Apart from funders’ perceived returns and their investment styles, the population, geographic area and disease burden of interest, the time frame in which returns are expected is an important defining component of the overall context. Priorities differ substantially if the overall context is one of great urgency to tackle a problem, or whether decisions are made on very long–term, strategic investments.

## CONCLUSIONS

The meeting with research funders organized by the WHO MNCAH department in March 2009 was exceptionally useful in understanding that funders certainly have their own views on what represents an attractive funding option. These views are not generalizable and may differ between categories of funders. Moreover, funders' perspectives are often quite different from those of researchers, or wider stakeholder groups. It is important to involve funders early in the process of setting research priorities, such as the CHNRI process, to encourage their ownership of the results. Funder–supported criteria must be taken into account, in addition to those preferred by the researchers and wider stakeholders. Otherwise, the outcomes of research prioritization exercises may have very limited impact on funders' decision making.

The key value of the CHNRI method to funders lies in its ability to transparently lay out the potential risks and benefits associated with investing in many competing research ideas, drawing on collective knowledge of the broad research community. Results of the CHNRI process represent an attempt on the part of researchers to communicate their views and opinions to funders in a way that is easily understood, transparent, replicable and intuitive. It provides useful additional information that funders may, or may not take into account when deciding on their own research agenda. From a methodological perspective, finding appropriate and effective ways of involving funders in future CHNRI exercises, communicating the outcomes clearly, and securing their commitment to acknowledge the results of the CHNRI process remain considerable challenges. An even greater challenge in future years will be to develop tools that can detect and evaluate the impact of CHNRI exercises on funder decision making and any change in funding priorities as a direct result of the CHNRI process. This should be particularly relevant to those who make decisions about investing public funds, whose primary agenda should be improving public health in the most cost–effective way – a target that CHNRI exercises should serve quite well.

## References

[1] Rudan I, Chopra M, Kapiriri L, Gibson J, Lansang MA, Carneiro I (2008). Setting priorities in global child health research investments: universal challenges and conceptual framework.. Croat Med J.

[2] Rudan I, Gibson JL, Ameratunga S, El Arifeen S, Bhutta ZA, Black M (2008). Setting priorities in global child health research investments: guidelines for implementation of the CHNRI Method.. Croat Med J.

[3] Rudan I, El Arifeen S, Bhutta ZA, Black RE, Brooks A, Chan KY (2011). Setting research priorities to reduce global mortality from childhood pneumonia by 2015.. PLoS Med.

[4] Fontaine O, Kosek M, Bhatnagar S, Boschi–Pinto C, Chan KY, Duggan C (2009). Setting research priorities to reduce global mortality from childhood diarrhoea by 2015.. PLoS Med.

[5] Bahl R, Martines J, Ali N, Bhan MK, Carlo W, Chan KY (2009). Research priorities to reduce global mortality from newborn infections by 2015.. Pediatr Infect Dis J.

[6] Lawn JE, Bahl R, Bergstrom S, Bhutta ZA, Darmstadt GL, Ellis M (2011). Setting research priorities to reduce almost one million deaths from birth asphyxia by 2015.. PLoS Med.

[7] Bahl R, Bhandari N, Bhutta ZA, Biloglav Z, Edmond K, Iyengar S (2012). Setting research priorities to reduce global mortality from low birth weight by 2015.. J Glob Health.

[8] World Health Organization. Consultation Proceedings: Identifying priorities for child health research to achieve Millennium Development Goal 4. DRAFT April 30, 2009. Geneva: WHO, 2009, pp. 1–29.

